# Impact of *Sinorhizobium meliloti* Exopolysaccharide on Adsorption and Aggregation in the Copper(II) Ions/Supporting Electrolyte/Kaolinite System

**DOI:** 10.3390/ma14081950

**Published:** 2021-04-13

**Authors:** Katarzyna Szewczuk-Karpisz, Agnieszka Tomczyk, Iwona Komaniecka, Adam Choma, Agnieszka Adamczuk, Weronika Sofińska-Chmiel

**Affiliations:** 1 Institute of Agrophysics, Polish Academy of Sciences, Doświadczalna 4, 20-290 Lublin, Poland; a.tomczyk@ipan.lublin.pl (A.T.); a.adamczuk@ipan.lublin.pl (A.A.); 2 Department of Genetics and Microbiology, Institute of Biological Sciences, Maria Curie-Sklodowska University, Akademicka 19, 20-033 Lublin, Poland; iwona.komaniecka@poczta.umcs.lublin.pl (I.K.); adam.choma@poczta.umcs.lublin.pl (A.C.); 3 Analytical Laboratory, Institute of Chemical Sciences, Faculty of Chemistry, Maria Curie-Sklodowska University, Maria Curie-Sklodowska Sq. 3, 20-031 Lublin, Poland; wschmiel@poczta.umcs.lublin.pl

**Keywords:** adsorption mechanism, single and mixed systems, surface charge density, zeta potential, CPS analysis, turbidimetry

## Abstract

To obtain insight into physicochemical interactions between Cu(II) ions, kaolinite, and exopolysaccharide (EPS) synthesized by *Sinorhizobium meliloti* Rm 1021 soil bacteria, an adsorption, electrokinetic, and aggregation study was performed in the selected systems. The obtained data showed that supporting electrolyte type affects both EPS and Cu(II) ions adsorption. For initial Cu(II) concentration 100 mg/L, 4.36 ± 0.25 mg/g (21.80 ± 1.00%) of the ions were adsorbed in 0.001 M NaCl and 3.76 ± 0.20 mg/g (18.80 ± 1.00%) in 0.001 M CaCl_2._ The experimental data were best fitted to the Langmuir model as well as pseudo second-order equation. The EPS adsorbed amount on kaolinite was higher in the CaCl_2_ electrolyte than in NaCl one. For an initial polymer concentration of 100 mg/L, the EPS adsorbed amount was 4.69 ± 0.08 mg/g (23.45 ± 0.40%) in 0.001 M NaCl and 5.26 ± 0.15 mg/g (26.32 ± 0.75%) in 0.001 M CaCl_2_. In the mixed system, regardless of electrolyte type, exopolysaccharide contributed to immobilization of higher amount of copper(II) ions on the clay mineral. Also, in the samples containing heavy metal ions and exopolysaccharide simultaneously, the aggregation of kaolinite particles was the strongest. The results presented in the paper may be very helpful in soil bioremediation, especially in the development of technologies reducing the mobility of heavy metals in the environment.

## 1. Introduction

The main aim of symbiosis between *Sinorhizobium* bacteria and Fabaceae plants is to enable the fixation of atmospheric nitrogen. At the beginning, soil bacteria penetrate into the plant through the root hairs. Then, an infection thread is created, which introduces the microorganisms deep into the plant′s tissue (primary cortex). The next step is formation of the root nodule, inside which the bacteria is transformed into bacteroids—the forms enable to atmospheric nitrogen fixation. Within the root nodules, the synthesis of proteins and enzymes necessary for N_2_ reduction occurs [1]. One of the most important factors in establishing symbiosis between bacteria and legume plant is exopolysaccharide (EPS)—a macromolecular compound produced by microorganisms and excreted into the environment [2].

Bacterial exopolysaccharides are large, long-chain biopolymers. They are composed of repeating oligosaccharidic subunits formed by carbohydrate monomers linked by α- and β-glycosidic bonds. EPS can be attached to the surface of cells forming an envelope (capsular polysaccharide—CPS) or can be secreted outside the cells as mucus (slime exopolysaccharide) [3]. Bacteria *Sinorhizobium meliloti* Rm 1021 synthesize two types of exopolysaccharide. The first, called EPS I, is a succinylglycan composed of octasaccharide subunits containing seven glucose molecules and one galactose molecule linked by β-1,3, β-1,4 and β-1,6 glycosidic bonds [4]. Succinylglycan is modified with succinyl, pyruvyl, and acetyl groups substituting the sugar backbone [5]. EPS I occurs in two fractions:

1. HMW (high molecular weight) characterized by molecular weight equal to 10^6^–10^7^ Da, as well as
2. LMW (low molecular weight) composed of monomers, dimers and trimers of octasaccharide subunits [4,5].

It has been shown that the LMW form is more biologically active and necessary for the development of effective nodules [2,6]. The second type of exopolysaccharide—EPS II, is a galactoglucan made of disaccharide basic units of acetylated glucose and galactose linked by β-1,3 and α-1,3 glycosidic bonds, substituted with a pyruvic acid and acetyl residue [4]. Exopolysaccharide plays a key role in the protection against pathogens (bacteriophages) as well as adverse environmental factors (extreme temperature or pH) [3]. What is more, it may influence the immobilization of heavy metals in the soil environment.

Soil pollution with heavy metals is mainly caused by emissions from rapidly expanding industrial areas, e.g., mine tailings, sewage sludge, pesticides, wastewater irrigation, atmospheric deposition and others [7]. Metal and non-metal ions can persist for a long time in the environment and pose a serious threat to animals and plants. These hazardous compounds decrease food quality (safety and marketability) via phytotoxicity and reduce land usability for agricultural production [8]. Most metals do not undergo microbial or chemical degradation [9]. Moreover, some of them inhibit the biodegradation of organic contaminants [7]. Heavy metals accumulated in soils can be released into other ecosystems and pollute surface and ground waters penetrating various food chains (soil-plant-human or soil-plant-animal-human) [8]. This migration occurs in the liquid and solid phases based on four major mechanisms: ion exchange, complexation, sorption and precipitation [10]. One approach for limiting mobility of pollutants is soil remediation using specific substances. The applied compounds reduce solubility and bioavailability of harmful elements as well as prevent their leaching to the aqueous ecosystems.

Recently, several scientists focused on the impact of polymeric substances, pH and temperature on the adsorption of metal or non-metal ions on clay minerals. For example, Fijałkowska et al. [11] found that lead(II) had a stronger affinity to the kaolinite surface in the presence of anionic polyacrylamide. The same team [12] observed that cationic polyacrylamide adsorbed on the kaolinite surface contributed to higher adsorption level of chromium(VI) ions. These phenomena were associated with the formation of polymer-heavy metal ion complexes. The ion adsorption on kaolinite was also dependent on the solution pH value. The lower pH value was, the larger adsorption capacity relative to Cr(VI) ions was observed. Jiang et al. [13] described the adsorption of copper(II) ions onto natural kaolinite clay. The adsorption capacity of the selected solid was higher at lower pH values. On the other hand, Wu et al. [14] showed that adsorptive abilities relative to Cu(II) ions of clay minerals were significantly improved due to their modification by humic acids.

The effect of bacterial exopolysaccharides on adsorptive properties of soil minerals is rarely described. Therefore, the authors investigated the physicochemical interactions between the exopolysaccharide *Sinorhizobium meliloti* Rm 1021, copper(II) ions and kaolinite. The performed study included:

1. adsorbed amount measurements of Cu(II) ions and EPS on kaolinite in the single and mixed systems (i.e., containing one or two adsorbates);
2. confirmation of Cu(II)/EPS adsorption using scanning electron microscope with EDS analyzer and Fourier transform infrared spectroscopy;
3. determination of surface charge density and zeta potential of kaolinite without and with Cu(II) ions and/or EPS;
4. aggregation study on kaolinite in the absence and presence of Cu(II) ions and/or EPS using turbidimetry and particle size analyzer (CPS).

The authors hypothesized that bacterial exopolysaccharide contributes to the adsorption of larger amount of copper(II) ions on the kaolinite surface and causes a strong aggregation of this clay mineral. The presented results can be very helpful in the development of innovative procedures for the treatment of soil or groundwater.

## 2. Materials and Methods

### 2.1. Materials

Kaolinite (CAS 1318-74-7), a clay mineral of 1:1 structure, delivered by Sigma-Aldrich (Saint Louis, MO, USA), was used in the study. Textural parameters of the selected solid were determined using nitrogen adsorption/desorption method (Micromeritics ASAP 2020 analyzer, Norcross, GA, USA). Kaolinite is an aluminosilicate of low porosity parameters. Its specific surface area equals 8.02 m^2^/g, while total pore volume (V_p_) is 0.0287 cm^3^/g. It contains two groups of mesopores of average diameters equal to 3.8 and 11.7 nm [12].

*Sinorhizobium meliloti* exopolysaccharide (EPS) and copper(II) ions were also used in the experiments. EPS was isolated at the Institute of Biological Sciences, UMCS, Lublin, Poland. The bulk EPS solution had a concentration of 500 mg/L, whereas the Cu(II) one—1000 mg/L. Copper(II) ions were applied in the form of CuCl_2_ (Sigma Aldrich, Saint Louis, MO, USA; CAS 7447-39-4). The pK_a_ value of *S. meliloti* exopolysaccharide, determined by potentiometric titration, was equal to 3.8 [15]. At pH 5, the dissociation degree (α) of selected polymer was 0.94.

The probes for adsorption, complexation, electrokinetic and aggregation studies were prepared using two types of supporting electrolyte, i.e., sodium chloride (NaCl) and calcium chloride (CaCl_2_) with the concentration of 0.001 M. The pH value of the examined samples was adjusted using 0.1 M HCl, 0.1 M NaOH and multifunction meter CX-505 (Elmetron, Zabrze, Poland).

### 2.2. Methods

#### 2.2.1. EPS Isolation

Exopolysaccharide of *Sinorhizobium meliloti* Rm 1021 was isolated according to the following procedure. At first, *S. meliloti* culture was carried on in 2-littre flasks containing 1 L 79CA medium containing glycerol and succinate as carbon sources [16], for 48 h, under continuous shaking (120 rpm), at 28 °C. Then, the bacteria were separated from the medium by centrifugation (30 min, 9000 rpm). The obtained supernatant was concentrated using vacuum evaporator and dialyzed by distilled water by 3 days. The EPS precipitation was performed using cold ethanol (3 volumes per 1 volume of EPS) and cooled (−20 °C, 24 h). The resulting EPS precipitate was sedimented by centrifugation (30 min, 4 °C, 8000 rpm), and obtained pellet was resuspended in deionised water and lyophilized.

#### 2.2.2. Adsorption Study

The adsorbed amount of EPS or copper(II) ions (Γ, mg/g) on the kaolinite surface was established based on the difference in the adsorbate concentration in the solution before (C_0_) and after (C_eq_) the adsorption according to the Equation (1) [17]:

(1) $$\Gamma =\frac{C_{\mathrm{ads}}\cdot V}{m}$$

where C_ads_ (mg/L) denotes the EPS/Cu(II) ions amount adsorbed on the surface (C_ads_ = C_0_-C_eq_), V (L) is the suspension volume, and m (g) is the adsorbent weight in the suspension.

The samples for the study were prepared by adding 0.05 g of kaolinite to the suspension containing supporting electrolyte as well as selected adsorbate (EPS or Cu(II)) or two adsorbates simultaneously. The optimal solid weight was selected based on the previous study on Cu(II) adsorption on various solid weights. After adjustment of the pH value to 5, the adsorption was started. The above pH value reflects the common conditions of Polish soils. Moreover, it was the highest pH value, at which copper occurs mainly as Cu^2+^ ions in the solution. The adsorption process was conducted for 24 h to ensure an equilibrium state in the studied systems. After its completion, the suspensions were filtered using paper filters and the obtained supernatants were analyzed to determine the copper(II) ions concentration by Mehling method [18] or EPS concentration by TOC analyzer (Multi N/C 2000, HT 1300, Analytik Jena, Jena, Germany). The adsorption isotherms of copper(II) ions were obtained for the initial Cu(II) concentrations in the range of 10–250 mg/L. These Cu(II) concentrations gave a plateau in adsorption isotherms. The obtained data were fitted to selected theoretical models, i.e., Langmuir (Equation (2)) and Freundlich (Equation (3)) [19]:

(2) $$q_{e}=\frac{q_{m}K_{L}C_{e}}{1+K_{L}C_{0}}$$

(3) $$q_{e}=K_{F}C_{e}^{1/n}$$

where K_F_ (mg/g(mg/L)^−1/nF^) is the Freundlich parameter, K_L_ (L/mg) is the Langmuir parameter, q_e_ (mg/g) is the equilibrium adsorption capacity, C_e_ (mg/L) is the equilibrium liquid phase concentration, q_m_ (mg/g) is the maximum adsorption capacity in Langmuir model, and n is the Freundlich constant related to adsorption intensity.

The specific surface area occupied by the copper ions (S_i_) at equilibrium was calculated from the following Equation:

(4) $$S_{i}=\frac{q_{m}{\mathrm{NA}}_{i}}{M}$$

where: N—Avogadro number, A_i_ (m^2^)—metal ion cross-sectional area, M (g)—ion molecular mass.

The obtained parameters showed the conditions, under which kaolinite is more effective adsorbent [20].

Due to the fact that the adsorption of macromolecular compound does not meet the assumptions of Langmuir and Freundlich models, the isotherms for exopolysaccharide were not obtained and modeled. The EPS adsorbed amounts were presented as histograms for the initial polymer concentration equal to 100 mg/L. In the mixed systems, i.e., containing Cu(II) ions and EPS in the same time, the initial concentration of two adsorbates was also 100 mg/L. The adsorption efficiency (E, %) was calculated using the Equation:

(5) $$E=\frac{C_{\mathrm{ads}}}{C_{0}}\cdot 100\%$$

where C_ads_—the concentration of adsorbed ions (mg/L), C_0_—the initial concentration (mg/L).

Kinetics of Cu(II) adsorption on kaolinite were determined in the probes containing 100 mg/L of heavy metal ions, at pH 5. The Cu(II) adsorbed amount was measured after adsorption process lasting 5, 10, 30, 60, 90, 120, 180, 240, 300 min. The obtained results were modeled using pseudo first-order Equation (6) and pseudo second-order Equation(7) [21,22]:

(6) $$\frac{{\mathrm{dq}}_{t}}{\mathrm{dt}}=k_{2}\left(q_{e}-q_{t}\right)$$

(7) $$\frac{{\mathrm{dq}}_{t}}{\mathrm{dt}}=k_{2}{\left(q_{e}-q_{t}\right)}^{2}$$

where: q_e_—the adsorbed amount at equilibrium [mg/g], q_t_—the adsorbed amount after time ′t′ (mg/g), k_1_ (1/min) and k_2_ (g/mg·min)—the equilibrium rate constants.

A single result was the average of three repetitions. The measurement error did not exceed 5%.

EPS adsorption on the kaolinite surface was also confirmed using Nicolet 6700 FTIR spectrometer (Thermo Scientific, Madison, WI, USA) equipped with Smart Orbit Diamond diamond attenuated reflectance (ATR) attachment. The spectra of kaolinite, EPS and kaolinite covered with EPS were recorded between 4000 and 400 cm^−1^ at 4 cm^−1^ intervals. Each spectrum was obtained from 128 scans and corrected with a linear baseline using OMNIC (v.8.2, Thermo Scientific, Madison, WI, USA).

#### 2.2.3. Complexes Formation Study

Complexes formation between exopolysaccharide and copper(II) ions was investigated analogously to the adsorption measurements. At the beginning, the probes containing 100 mg/L of EPS, 100 mg/L of Cu(II) ions, and 0.001 M of supporting electrolyte were prepared. Then, the pH value of the solutions was adjusted to 5. After 24 h continuous shaking, the samples were centrifuged 3 times and the obtained supernatants were analyzed towards determination of copper ions concentration in them [18]. A single result was the average of three repetitions. The measurement error did not exceed 5%.

#### 2.2.4. Surface Charge Determination

Surface charge density (σ_0_) of kaolinite in the absence and presence of copper(II) ions and *S. meliloti* exopolysaccharide was determined using potentiometric titration (Titrino 702 SM burette, Metrohm, Herisau, Switzerland). The following systems were titrated:

1. supporting electrolyte (0.001 M NaCl or CaCl2);
2. supporting electrolyte + kaolinite
3. supporting electrolyte + kaolinite + Cu(II) ions;
4. supporting electrolyte + kaolinite + EPS;
5. supporting electrolyte + kaolinite + Cu(II) ions + EPS.

The concentration of adsorbates was equal to 100 mg/L. The mass of kaolinite using to the suspension preparation was 0.1 g. The titrations of the systems without heavy metal were carried out in the pH range 3.5–10. In turn, in order to avoid the hydroxide precipitation, the measurements of the suspensions with Cu(II) ions were performed in the pH range 3.5–6. The σ_0_ parameter was calculated using the following formula [23]:

(8) $$\sigma_{0}=\frac{∆V\cdot C\cdot F}{m\cdot S_{BET}}$$

where: ΔV (mL)—the difference in the base volume added to the suspension and the supporting electrolyte that leads to the specific pH value, C (mol/L)—the base concentration, F—the Faraday constant, m (g)—the solid mass in the suspension, S_BET_ (m^2^/g)—the solid surface area.

#### 2.2.5. Zeta Potential Calculation

Electrophoretic mobility of the kaolinite particles without and with Cu(II) ions/exopolysaccharide was determined using microelectrophoresis phenomenon, i.e., zetasizer NanoZS (Malvern Instruments, Worcestershire, UK). Based on obtained results, zeta potential (ζ) of the selected solid was calculated by the computer software [24]. The suspensions for the experiment were prepared by adding 0.2 g of kaolinite to 200 mL of the solution containing supporting electrolyte (0.001 M NaCl or CaCl_2_) without or with Cu(II) ions/exopolysaccharide. Such suspensions were divided into several parts of different pH values (3–9 in the case of the systems without Cu(II) ions, 3–6 in the case of the systems containing heavy metal ions). The concentration of Cu(II) ions and exopolysaccharide used in the suspension preparation was equal to 100 mg/L.

#### 2.2.6. Aggregation study

The aggregation of kaolinite particles was established based on the turbidimetric measurements (Hach 2100AN turbidimeter, Omc Envag, Warsaw, Poland) as well as CPS analyses (DC24000 analyzer, CPS Instruments, Anaheim, CA, USA). The samples were prepared by addition 0.02 g of kaolinite to supporting electrolyte (0.001 M NaCl or CaCl_2_). After 3 min sonication, the one or two adsorbates (Cu(II) ions or exopolysaccharide with the concentration of 100 mg/L) were added (the final volume of the sample was 20 mL). After pH adjustment to the value of 5, the CPS or turbidimetric analysis was started. A single measurement of system turbidity lasted 60 min. CPS analyses were performed using sucrose gradient, prepared by mixing 8% and 24% solutions. The disc speed was equal to 2500 rpm. The particle/aggregate size was measured in the diameter range of 0.11–5 μm.

## 3. Results and Discussion

### 3.1. Mechanism of Copper(II) Ions Adsorption on Kaolinite

**Figure 1:**
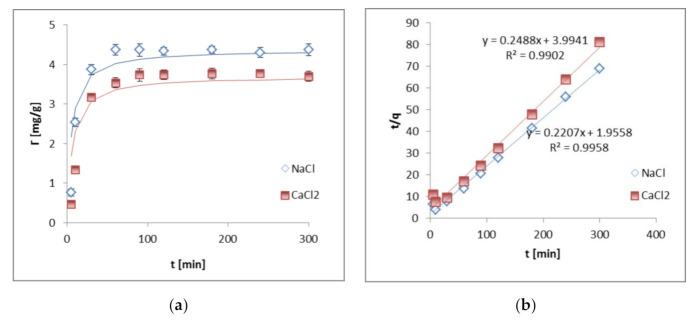

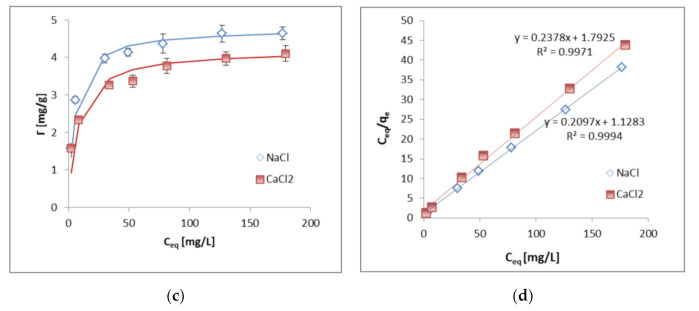
Copper(II) ion adsorption kinetics on kaolinite: data (points) and pseudo second-order fitting (lines) in the NaCl or CaCl_2_ solution (**a**) as well as the obtained linear forms of PSO Equation (**b**); Adsorption isotherms of copper(II) ions on kaolinite: data (points) and Langmuir fitting (lines) (**c**) as well as their linear forms (**d**); at pH 5, in the NaCl or CaCl_2_ solution.

The results of Cu(II) adsorption kinetics on kaolinite, presented in Figure 1a, showed that equilibrium in the examined systems was reached after 90 min in both supporting electrolyte types.

The obtained data were better fitted to the pseudo second-order (PSO) equation than to the pseudo first-order (PFO) one (Figure 1a, Table 1).

The calculated R^2^ values for PSO equal 0.995 in NaCl and 0.990 in CaCl_2_, whereas the R^2^ values for PFO were 0.368 in NaCl and 0.403 in CaCl_2_. This indicates that Cu(II) adsorption on kaolinite involves electron sharing or exchange between adsorbent and adsorbate (chemisorption) [25].

The obtained adsorption isotherms of Cu(II) ions on the kaolinite surface, measured for two various supporting electrolytes (NaCl or CaCl_2_) are presented in Figure 1c. The calculated isotherm parameters indicated that obtained experimental data are better fitted to Langmuir model (Table 1). In 0.001 M NaCl solution, the R^2^ value was 0.994, whereas in 0.001 M CaCl_2_—0.997. Such a good fitting to the Langmuir model indicated that heavy metal ions formed monolayer on the kaolinite surface characterized by uniform adsorption energy [17,26,27]. At pH 5, copper(II) ions occur mainly in the form of Cu^2+^. The concentration of Cu(OH)^+^ species in the system is relatively low [28]. In the examined systems, Cu^2+^ ions interact with one or two adjacent surface groups according to reactions:

(9) $$-{\mathrm{SO}}^{-}+{\mathrm{Cu}}^{2+}\;\to \;-\mathrm{SO}-{\mathrm{Cu}}^{+}$$

(10) $$2\left(-{\mathrm{SO}}^{-}\right)+{\;\mathrm{Cu}}^{2+}\to 2\left(-\mathrm{SO}\right)\mathrm{Cu}$$

So, the adsorption layer formed on kaolinite may be positively charged and repel other heavy metal ions [28,29,30]. Due to this fact, the Cu(II) adsorbed amount on the selected clay mineral does not exceed 22%. In addition to electrostatic interactions, Cu(II) adsorption on kaolinite may be connected by other mechanisms, i.e., ion exchange, surface complexation and van der Waals attractions [31]. The previous study on the adsorption of lead(II) and chromium(VI) ions on kaolinite also indicated the best fitting of adsorption isotherm data to Langmuir model [11,12,32].

Potentiometric titration of kaolinite showed that the point of zero charge (pH_pzc_) of the used clay mineral, determined in 0.001 M NaCl, is about 3.7. In turn, in the solution of 0.001 M CaCl_2_ this parameter is about 4.1. This means that at pH 5, at which adsorption measurements were carried out, the surface of kaolinite particles has a slight negative charge. In the 0.001 M NaCl solution the adsorbent surface charge equals –3.2 μC/cm^2^, whereas in 0.001 M CaCl_2_ the σ_0_ parameter is −1.2 μC/cm^2^. Thanks to such adsorbent surface charge, copper(II) ions have a clear affinity to the solid surface (electrostatic attraction). Owing to stronger attractive forces occurring between heavy metal cations and kaolinite particles in the NaCl supporting electrolyte, the Cu(II) adsorbed amount was higher in this solution (compared to CaCl_2_ one). For initial Cu(II) concentration 100 mg/L, in 0.001 M NaCl 4.36 ± 0.25 mg/g (21.80 ± 1.00%) of the ions were adsorbed, whereas in 0.001 M CaCl_2_—3.76 ± 0.20 mg/g (18.80 ± 1.00%). The observed adsorbed amounts may be also associated with competition between bidentate cations (calcium and copper ones) present in the examined systems. The specific surface area occupied by the copper ions (S_i_) at equilibrium for 0.001 M NaCl was 0.91 m^2^/g, whereas for 0.001 M CaCl_2_—0.62 m^2^/g. The cross-sectional area of Cu^2+^ ions is 1.58 Å^2^ [20].

**Figure 2:**
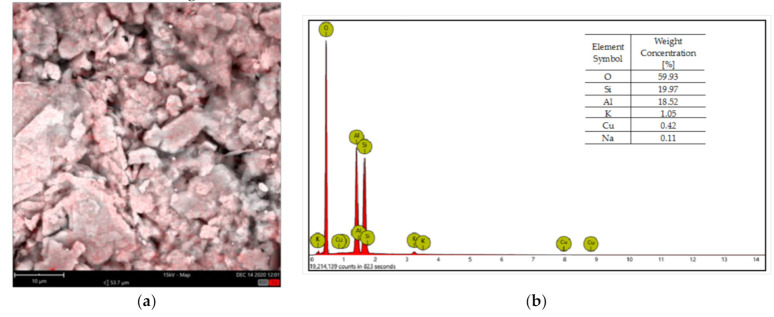
SEM-EDS maps (**a**) and elemental composition (**b**) of kaolinite after the Cu(II) adsorption (copper is marked in red).

The Cu(II) adsorption on kaolinite was confirmed using scanning electron microscope with EDS analyzer. The obtained map and element composition are presented in Figure 2.

### 3.2. Mechanism of Exopolysaccharide Adsorption on the Kaolinite Surface

**Figure 3:**
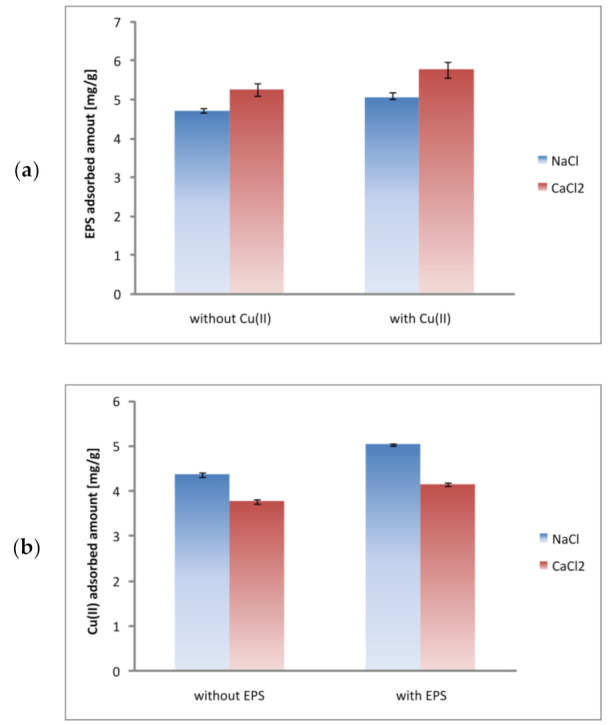
EPS adsorbed amount [mg/g] without and with Cu(II) ions (100 mg/L) (**a**) as well as Cu(II) adsorbed amount [mg/g] without and with EPS (100 mg/L) (**b**) in various supporting electrolytes (0.001 M NaCl or CaCl_2_).

The exopolysaccharide adsorbed amounts noted on the kaolinite surface are presented in Figure 3a.

The data obtained for EPS were not fitted to Langmuir nor Freundlich Equations because the adsorption of macromolecular compounds does not meet the assumptions of selected theoretical models. The results, presented as a histogram, showed that exopolysaccharide macromolecules were adsorbed on the kaolinite surface at pH 5. However, the EPS adsorbed amounts were slightly higher in the 0.001 M CaCl_2_ solution (compared to 0.001 M NaCl one). For initial polymer concentration 100 mg/L, in 0.001 M NaCl the EPS adsorbed amount was 4.69 ± 0.08 mg/g (23.45 ± 0.40%), whereas in 0.001 M CaCl_2_—5.26 ± 0.15 mg/g (26.32 ± 0.75%). Higher values observed in the system containing calcium chloride is dictated by more coiled structure of macromolecules in comparison to that assumed in the presence of sodium chloride. The previous studies indicated that, at pH 5, the dissociation degree (α) of exopolysaccharide equals 0.94. Thus, under these pH conditions, 94% of carboxylic groups are dissociated. The polymer segments containing negatively charged moieties repel each other, and as a consequence the EPS macromolecules have strongly expanded conformation. Calcium ions, owing to their bidentate character, may interact with two functional groups of the polymer chains. As a result, intra-molecular complexes, having more coiled conformation than uncomplexed macromolecules, are formed. During the adsorption, the EPS-Ca(II) complexes take a smaller part of adsorbent surface, so the adsorbed amount of the selected polymer may be larger. The formation of EPS-Na(I) complexes also occurs in the systems containing NaCl supporting electrolyte. However, this phenomenon does not change the conformation of polymer chains.

**Figure 4:**
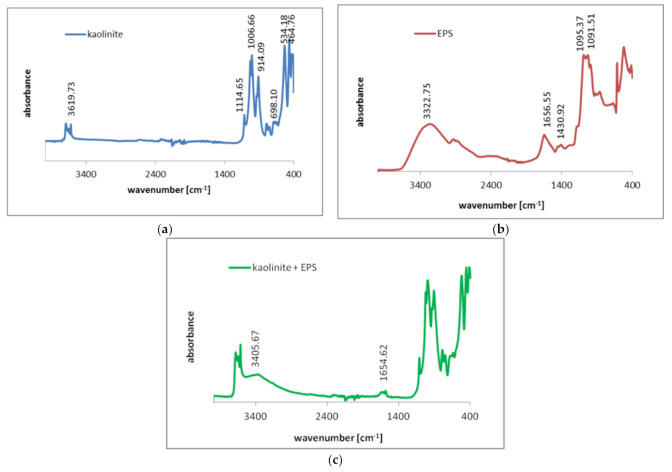
FTIR spectra of kaolinite (**a**), EPS (**b**) and kaolinite covered with EPS (**c**).

The exopolysaccharide adsorption on the kaolinite surface of slight negative charge is mainly based on the formation of hydrogen bonds between adsorbent and adsorbate functional groups. This mechanism is very possible in the presence of repulsive electrostatic forces acting between solid particles and polymer chains. The adsorption of *S. meliloti* exopolysaccharide was confirmed using Fourier transform infrared spectroscopy. The obtained spectra for kaolinite, EPS, as well as kaolinite covered with EPS are presented in Figure 4.

The kaolinite spectrum contained band specific for this clay mineral. There were bands at:

1. 464.76 and 534.18 cm^−1^ (corresponding with Si-O stretching vibrations);
2. 698.10 cm^−1^ (attributed to Mg/Al–OH vibrations);
3. 800.31 cm^−1^ (the Si–O–Al group deformation);
4. 914.09 cm^−1^ (the OH bending vibrations of Al–OH);
5. 1006.66 and 1114.65 cm^−1^ (the Si–O stretching) [33,34].

The spectrum of exopolysaccharide was composed of the following bands at: (1) 1091.51 and 1095.37 cm^−1^ (corresponding with C–H bending vibrations); (2) 1430.92 cm^−1^ (attributed to CH_2_ symmetrical stretching); (3) 1656.55 cm^−1^ (the –C=C stretching vibrations); (4) 3322.75 cm^−1^ (the C=C–H group stretching) [35]. The spectrum of the kaolinite particles obtained after EPS adsorption contained two specific bands, typical for selected macromolecular compound. They were at: (1) 1654.62 cm^−1^ (corresponding with –C=C group stretching) and (2) 3405 cm^−1^ (attributed to C=C–H group stretching). These bands clearly confirmed the exopolysaccharide presence on the solid surface.

### 3.3. Cu(II) and EPS Immobilization on Kaolinite in the Mixed Systems

The adsorbed amounts of exopolysaccharide and copper(II) ions noted in the mixed systems, i.e., containing polymer and heavy metal ions simultaneously, are presented in Figure 3a,b. The experiments showed that EPS contributed to slight increase in the Cu(II) adsorbed amount on the kaolinite surface (Figure 3b). This means that in the *S. meliloti* exopolysaccharide presence, the immobilization of copper(II) ions on the clay mineral is higher. For initial Cu(II) concentration equal to 100 mg/L, in the 0.001 M NaCl solution, the Cu(II) adsorbed amount was 5.03 ± 0.17 mg/g (25.15 ± 0.85%), in turn in the 0.001 M CaCl_2_ solution—4.15 ± 0.15 mg/g (20.75 ± 0.75%). The study on EPS-Cu(II) complexation indicated that, for initial Cu(II) concentration equal to 100 mg/L, 10.76 ± 0.20 mg/L (in the NaCl system) and 2.06 ± 0.05 mg/L (in CaCl_2_) of Cu(II) ions are bounded with the EPS macromolecules. In other words, 10.76 ± 0.20% and 2.06 ± 0.05% of heavy metal ions in NaCl and CaCl_2_, respectively, are arranged in the formation of complexes with macromolecular compound. Negative EPS macromolecules attract a certain amount of cations electrostatically. There may exist monocarboxylic and dicarboxylic complexes in the system [36]:

(11) $$\left[{\mathrm{Cu}}_{t}\right]=\left[{\mathrm{Cu}}^{2+}\right]+\;\left[{\mathrm{CuA}}^{+}\right]+\;\left[{\mathrm{CuA}}_{2}\right]$$

(12) $$\left[A_{t}\right]=\left[A^{-}\right]+\;\left[\mathrm{HA}\right]+\;\left[{\mathrm{CuA}}^{+}\right]+\;2\left[{\mathrm{CuA}}_{2}\right]$$

where: [Cu_t_], [A_t_]—the total concentrations of copper(II) ions and carboxylic groups, [CuA^+^], [CuA_2_], [A^−^]—the concentrations of monocarboxylic copper(II) complexes, dicarboxylic copper(II) complexes and non-complexed carboxylic groups, respectively.

The bond between metal ion and polymer functional group may be also realized through coordinated bond (chelate complexes) or ionic bond (molecular complex) [37]. These EPS-Cu(II) complexes (in most of intra-molecular character) are adsorbed on the kaolinite surface together with free (uncomplexed) Cu(II) ions and, as a result, the heavy metal adsorbed amount increases. Owing to this phenomenon, the mobility and bioavailability of copper(II) ions to plants and animals in the soil environment enriched with *Sinorhizobium meliloti* exopolysaccharide are lower than in the soil without the selected EPS.

On the other hand, the Cu(II) ions presence in the examined system increases the exopolysaccharide adsorbed amount on kaolinite surface (Figure 3a). For initial EPS concentration equal to 100 mg/L, in 0.001 M NaCl, the EPS adsorbed amount was 5.04 ± 0.16 mg/g (25.20 ± 0.80%), in turn in 0.001 M CaCl_2_—5.76 ± 0.20 mg/g (28.80 ± 1.00%). As it was mentioned above, bidentate ions contribute to more coiled conformation of polymer chains by formation of intra-molecular complexes. Moreover, the concentration of copper(II) ions in the system is sufficiently high that they can participate in inter-molecular complexation. These complexes are created between adsorbed and non-adsorbed polymer chains and, as a result, the second adsorption layer of exopolysaccharide can be even formed.

### 3.4. Copper(II) ions and Exopolysaccharide Effect on Kaolinite Surface Charge Density

**Figure 5:**
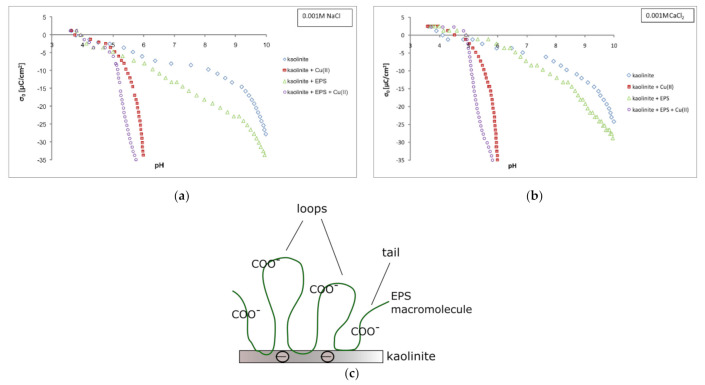
Surface charge density (σ_0_) of kaolinite in the absence and presence of Cu(II) ions and/or EPS (100 mg/L) in 0.001 M NaCl (**a**) and 0.001M CaCl_2_ (**b**) as well as structures of EPS macromolecules on the kaolinite surface at pH 5 (**c**).

Potentiometric titration indicated that the point of zero charge (pH_pzc_) of kaolinite in 0.001 M NaCl equalled 3.7, whereas in 0.001 M CaCl_2_—4.1. At pHs < pH_pzc_ the solid surface charge is positive, in turn at pHs > pH_pzc_—negative. The results of conducted potentiometric titrations, i.e., the dependency of kaolinite surface charge density on the system pH value, are presented in Figure 5a,b.

The value of pH_pzc_ in NaCl and CaCl_2_ solution differs due to various adsorption mechanism of Ca^2+^ and Na^+^ ions on kaolinite. The cations of the 1st group of metals and several monovalent anions (chloride, nitrate) show non-specific adsorption (based on electrostatic attraction) and, as a consequence, they do not affect the pH_pzc_ parameter. The ions adsorbed non-specifically are located either in the diffuse Gouy–Chapman layer or at the outer Helmholz plane and separated from the surface by at least one water molecule [38,39,40]. The ions of calcium, i.e., of the metal from 2nd group, are also adsorbed in a specific way (based on chemical attraction (covalent or coordinate)). They are located in the inner Helmholz layer and may shift the pH_pzc_ value significantly [40].

In the presence of copper(II) ions, regardless of supporting electrolyte type, the absolute values of negative kaolinite surface charge were higher than those noted without heavy metal ions. These changes in the σ_0_ parameter were dictated by the following reactions between hydroxyl surface groups and Cu(II) ions [41]:

(13) $$\equiv \mathrm{SOH}+{\mathrm{Cu}}^{2+}⇌\;\equiv {\mathrm{SO}}^{-}{\mathrm{Cu}}^{2+}+H^{+}$$

(14) $$2\left(\equiv \mathrm{SOH}\right)+{\mathrm{Cu}}^{2+}⇌{\left(\equiv \mathrm{SO}\right)}_{2}{\mathrm{Cu}}^{2+}+2H^{+}$$

(15) $$\equiv \mathrm{SOH}+{\mathrm{Cu}}^{2+}+H_{2}O⇌\;\equiv {\mathrm{SO}}^{-}{\mathrm{CuOH}}^{+}+2H^{+}$$

The exopolysaccharide addition to the examined suspensions also contributed to higher absolute values of negative kaolinite surface charge (compared to the solid surface charge noted without any adsorbates). This phenomenon, identical in two studied supporting electrolytes, was induced by dissociated carboxylic groups located in the ′loops′ and ′tails′ structures formed by exopolysaccharide on the kaolinite surface (Figure 5c). Due to negative charge of particles, the contact between them and EPS macromolecules is minimal. The polymer chains interact only with several surface groups; the vast majority of polymer segments is directed toward the solution. Thus, the number of carboxylic groups located in ′loops′ and ′tails′ structures is significantly higher than the number of the same moieties placed close to the kaolinite surface. ′Loops′ and ′tails′ are structures typical for macromolecular compounds adsorbed on the solid surface. Owing to them, one polymer chain can interact with several active sites on the adsorbent. ′Loops′ and ′tails′ were also observed in the following systems: poly(acrylic acid)/chromium(III) oxide [42], exopolysaccharide/silica [43], polyacrylamide/kaolinite [11].

In the mixed systems containing copper(II) ions and exopolysaccharide, in the case of both supporting electrolytes, the increase in absolute values of negative kaolinite surface charge was also noted. Probably, this is a result of combined impacts of heavy metal ions and exopolysaccharide adsorbed on the kaolinite particles.

### 3.5. Exopolysaccharide and Copper(II) Ions Effect on Potential of Kaolinite Slipping Plane Area

**Figure 6:**
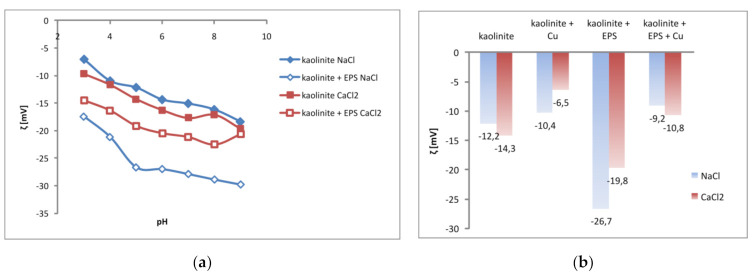
Zeta potential (ζ) of kaolinite in the absence and presence of EPS (100 mg/L) in the NaCl or CaCl_2_ solution (**a**), as well as in the systems containing only Cu(II) ions (100 mg/L) or Cu(II) ions and EPS simultaneously (**b**).

The obtained zeta potential values of kaolinite, in the absence and presence of copper(II) ions and exopolysaccharide, are presented in Figure 6.

Isoelectric point (pH_iep_) of kaolinite was not determined in the examined pH range of 3–9. This means that the value of this parameter, both in NaCl and CaCl_2_ solution, is lower than 3. At the examined pH values (3–9), electrokinetic potential of kaolinite was negative, which indicated that negatively charged groups were dominant in the clay slipping plane area. The absolute values of negative zeta potential of kaolinite calculated for NaCl were minimally lower than those calculated for CaCl_2_.

Regardless of supporting electrolyte type, *S. meliloti* exopolysaccharide caused the increase in absolute values of negative kaolinite zeta potential (Figure 6a). This observation is associated with the slipping plane offset by the adsorbed EPS macromolecules, especially by ′loops′ and ′tails′ of considerable length. Higher absolute values of negative zeta potential are also dictated by dissociated carboxylic groups of polymer chains located in the slipping plane area.

The copper(II) ions presence in the kaolinite solution contributed to decrease in absolute values of negative zeta potential (compared to the suspension without adsorbates, Figure 6b). These changes are mainly associated with hydrated Cu(II) ions in the outer Helmholtz layer. They are adsorbed non-specifically based on electrostatic interactions [44]. The presence of both copper(II) ions and exopolysaccharide in the kaolinite suspension resulted also in a reduction in absolute values of negative zeta potential.

### 3.6. Different Impacts of Exopolysaccharide and Copper(II) Ions on Kaolinite Aggregation

**Figure 7:**
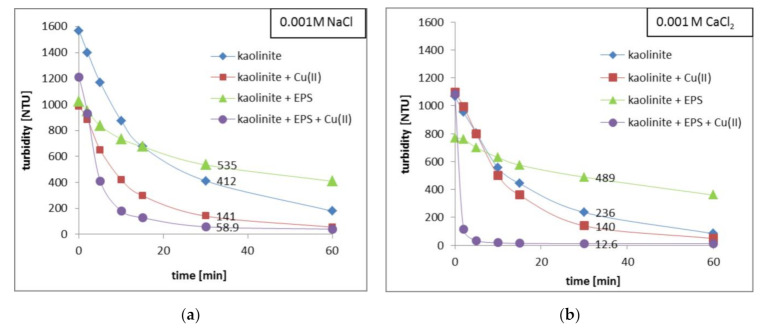
Turbidity changes in kaolinite suspension without and with Cu(II) ions and/or EPS (100 mg/L) in 0.001 M NaCl (**a**) and 0.001M CaCl_2_ (**b**).

**Figure 8:**
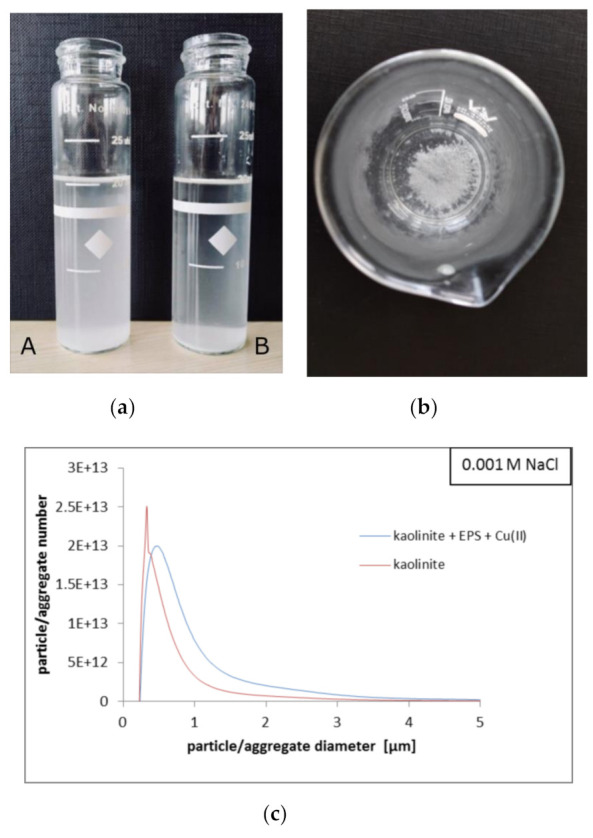
Photo of kaolinite suspension without(A) and with(B) EPS/Cu(II) ions after 15 min (**a**); photo of flocs formed in the kaolinite/Cu(II)/EPS/CaCl_2_ system (**b**); particle/aggregate number vs. particle/aggregate diameter measured for kaolinite suspension without or with Cu(II) ions and EPS (100 mg/L) in 0.001 M NaCl (**c**).

The results of turbidimetry and particle size (CPS) analyses are presented in Figure 7 and Figure 8 and Table 2.

They showed that copper(II) ions and *S. meliloti* exopolysaccharide, added to the system separately or simultaneously, influenced the kaolinite aggregation in a different way. However, the changes noted after addition of selected adsorbates were similar for both supporting electrolytes.

The strongest aggregation of kaolinite was observed in the mixed systems containing copper(II) ions and exopolysaccharide together. After 30 min, the turbidity of the kaolinite/Cu(II)/EPS/NaCl system was 58.9 NTU, whereas that of kaolinite/Cu(II)/EPS/CaCl_2_ equalled only 12.6 NTU (Figure 7). For comparison, the turbidity of the kaolinite suspension without any adsorbate in NaCl after selected time was 412 NTU, in turn in CaCl_2_—236 NTU. This indicated that in the simultaneous presence of heavy metal ions and polymer, the largest, quickly sedimenting aggregates are formed. Under these conditions, the clarification of the examined suspension is the fastest (Figure 8a). The CPS analyses showed that in the kaolinite suspension in NaCl the particles/aggregates of diameter 0.329 μm prevailed, whereas in CaCl_2_—those of 0.327 μm were the most numerous. After addition of Cu(II) ions and EPS, in the system containing NaCl the aggregates of diameter equal to 0.448 μm were dominant. What is more, in the kaolinite/Cu(II)/EPS/CaCl_2_ system the formed aggregates were so large that they exceeded the measuring range of the CPS device, i.e., 0.11–5 μm (Figure 8b, Table 2). Stronger aggregation in the systems containing CaCl_2_ as a supporting electrolyte is dictated by bridging properties of calcium(II) cations [45].

Copper(II) ions, added to the kaolinite suspension separately, also contributed to stronger aggregation of solid particles, but to a lesser extent than Cu(II) ions and exopolysaccharide together. After 30 min the turbidity of the kaolinite/Cu(II)/NaCl system was 141 NTU, whereas that of kaolinite/Cu(II)/CaCl_2_ one—140 NTU.

On the other hand, the addition of only exopolysaccharide, regardless of supporting electrolyte type, contributed to higher turbidity of the kaolinite suspension. After 30 min the turbidity of the kaolinite/NaCl system was 535 NTU, whereas that of kaolinite/CaCl_2_ one—489 NTU. This means that the EPS presence limits the mutual contact between solid particles and hiders the aggregate formation.

The above phenomena are associated with several mechanisms. The reduction in suspension stability observed after the addition of copper(II) ions is mainly dictated by neutralization of negative surface charge of solid particles by adsorbed heavy metal cations. Under these conditions the absolute values of kaolinite zeta potential are the lowest among all tested systems, which also favors the solid aggregation (Figure 6b). In turn, the exopolysaccharide presence in the kaolinite suspension contributes to higher system stability based on electrosteric interactions [46]. The EPS macromolecules non-adsorbed or adsorbed on the solid surface create a steric barrier between the particles. What is more, carboxylic groups present in the polymer chains repel each other electrostatically. In this case, the absolute values of kaolinite zeta potential are the highest among all examined suspensions, which is equivalent to strong limitation in solid aggregation (Figure 6a). Clear system destabilization resulted in formation of big flocs (Figure 8b), noted in the mixed systems containing two adsorbate types, is mainly dictated by described-above creation of inter-molecular complexes between adsorbed polymer chains and copper(II) ions. Owing to this mechanism, the solid particles are bound together by specific bridges.

## 4. Conclusions

The performed experiments allowed to define the nature of physicochemical interactions between copper(II) ions, *S. meliloti* exopolysaccharide and kaolinite. They enable the formulation of following conclusions:

1. Due to the competition between bidentate cations for access to the adsorbent surface, the Cu(II) adsorbed amount on kaolinite in CaCl_2_ solution was lower than that in NaCl supporting electrolyte. For initial Cu(II) concentration 100 mg/L, in 0.001 M NaCl 4.36 ± 0.25 mg/g (21.80 ± 1.00%) of the ions was adsorbed, whereas in 0.001 M CaCl_2_—3.76 ± 0.20 mg/g (18.80 ± 1.00%).
2. Regardless of electrolyte type, the experimental data of Cu(II) adsorption isotherms were best fitted to Langmuir model. This indicated, inter alia, that heavy metal ions formed monolayer on the clay mineral surface characterized by uniform adsorption energy. The kinetics of the Cu(II) adsorption on kaolinite were best fitted to the pseudo second-order equation, which meant that the examined phenomenon involved chemisorption.
3. The adsorbed amount of exopolysaccharide on kaolinite was larger in the CaCl_2_ electrolyte than in NaCl one. For initial polymer concentration 100 mg/L, in 0.001 M NaCl the EPS adsorbed amount was 4.69 ± 0.08 mg/g (23.45 ± 0.40%), whereas in 0.001 M CaCl_2_—5.26 ± 0.15 mg/g (26.32 ± 0.75%). This is a result of the formation of intra-molecular EPS-Ca(II) complexes having more coiled conformation than uncomplexed macromolecules.
4. In the mixed system, exopolysaccharide contributed to higher adsorption level of copper(II) ions. What is more, heavy metal ions also make the exopolysaccharide adsorbed amount on the clay mineral larger. The above observations are mainly associated with Cu(II) ions-EPS complexation.
5. Adsorption of copper(II) ions and/or *S. meliloti* exopolysaccharide affected the structure of electrical double layer of kaolinite, significantly. There are clear changes in zeta potential values and surface charge of clay mineral.
6. In the samples containing Cu(II) ions and exopolysaccharide simultaneously, the kaolinite aggregation was the strongest. This is mainly dictated by the formation of specific bridges (consisted of polymer chains and heavy metal ions) between solid particles.
7. In the environment contaminated with copper(II) ions, *Sinorhizobium meliloti* exopolysaccharide contributes to a higher immobilization of selected heavy metal as well as stronger aggregation of clay minerals, which is highly desirable in soil remediation technologies.

## Figures and Tables

**Table 1:** Kinetics and isotherm parameters acquired from selected models of Cu(II) adsorption on kaolinite, at pH 5, in the NaCl or CaCl_2_ solution.

		Pseudo I-Order Equation			Pseudo II-Order Equation		
System		q_e_ [mg/g]	k_1_ [1/min]	R^2^	q_e_ [mg/g]	k_2_ [g/(mg·min)]	R^2^
Kinetics	NaCl	3.136	0.0382	0.368	4.531	0.025	0.995
	CaCl_2_	1.751	0.060	0.403	4.019	0.015	0.990
		Langmuir Model			Freundlich Model		
System		q_m_ [mg/g]	K_L_ [L/mg]	R^2^	n	K_F_ [mg/g (mg/L)^−1/nF^]	R^2^
Isotherms	NaCl	4.768	0.186	0.999	4.454	0.218	0.887
	CaCl_2_	4.204	0.132	0.997	4.950	0.260	0.947

**Table 2:** Average diameter [μm] of particles/aggregates in kaolinite suspension prepared using two different supporting electrolytes; the CPS analyses were performed in the diameter range from 0.11 to 5 μm.

System		(µm)
Kaolinite	NaCl	0.329
Kaolinite + Cu(II)	–	0.401
Kaolinite + EPS + Cu(II)	–	0.488
Kaolinite	CaCl_2_	0.327
Kaolinite + Cu(II)	–	0.371
Kaolinite + EPS + Cu(II)	–	0.241

## Data Availability

The reported results can be found at the Institute of Agrophysics, PAS, Lublin, Poland.
